# Case (of Fever?)

**Published:** 1827-12

**Authors:** George Gregory

**Affiliations:** senior Physician to the St. George's and St. James's General Dispensary.


*Case (of Fever?).* By George Gregory, M.D. senior Physician to the St. George's and St. James's General Dispensary.

In the forenoon of Monday, October 13th, 1823, I was called to give my professional advice to Adele Anselle, a young woman, twenty-one years of age, lately arrived from France, residing with Madame M., an embroiderer and dress-maker, at No. 14, Warwick-street, Golden-square. The symptoms were as follows:—High fever, hot skin, pulse 120, strong and full; great expression of anxiety in the countenance; no urgent pain. When pressed to describe her sensations, she said she had *mal au cœur* (sickness at heart,) and an excessively bad taste in the mouth. This latter symptom appeared to predominate over the former, and indeed over every other. She was in bed, and further complained of excessive weakness. She had been observed by those around her drooping during the whole of the preceding week, but she never made much complaint, and she persevered in doing a little work. The day previous to my seeing her, she for the first time kept her bed.

Finding that she had of her own accord taken an emetic, which had acted effectually, but without benefit, I made some further inquiries with the view of directing my practice. The result was as follows:—Bowels not open, but no fulness or tenderness of the abdomen; draws her breath deep without inconvenience; no sense of fluttering at the heart; occasionally she has a short cough, but she does not complain of it; tongue loaded; no delirium. I formed no distinct notion of the disease, but thought it advisable to take away blood. A vein was opened freely, but the blood would not flow. She became pale in the face, and faint.

A purgative ordered.

Monday evening.—Worse. Fever runs high. Complains of an undescribable feeling about the sternum. Something, she says, seems to come up into her mouth, and causes a horribly bad taste. Pulse very full and active; great restlessness; general distress, urgent in the extreme. I directed Mr. Harding, apothecary to the Dispensary, to bleed her, if possible. He got about ten ounces, when she fainted.

Tuesday morning.—Rather better. Passed a tolerable night. Pain of the legs was the principal symptom complained of at this visit.

Saline medicines ordered.

Tuesday evening.—All the former distressing symptoms are returned.

A purgative draught, to be followed by a diaphoretic julep.

Wednesday.—Uneasiness today very great, and is now decidedly referred to the region of the heart. Pulse very bounding and irregular. Another attempt made to bleed her, but the blood would not flow from the arm. She was cupped on the chest to twelve ounces, with some degree of relief.

Wednesday evening.—Distress more urgent. Again I tried to bleed her, but only an ounce or two of blood flowed. Leeches were directed, and fomentations. At this visit I had the assistance of Mr. Callow, surgeon of the 84th Regiment.

Thursday morning.—Mr. Corbett, surgeon, of Titchborne-street, accompanied me. The distress of the patient was excessive. She earnestly prayed for some relief. The pulse was very intermittent, but it bounded against the finger with singular force. We determined to put her into a warm bath, and in that situation to bleed her. However, before we could get things ready for this latter operation, she fell into a deep and long faint. On her recovery from that state, some doses of Calomel were given.

Thursday evening.—Countenance expressive of the most urgent distress. She is tossing about the bed, but, when asked, says she has no positive local pain. She distinctly states, however, that her respiration is not free. Late at night, finding her distressed condition even worse, I determined to try what a long-continued faint would do. I put on twelve leeches to the pit of the stomach, and immediately afterwards bled her in the arm. By these means I produced a long-continued faint of three hours and a half, during which time I fully expected to see her die. She recovered, however, and passed the rest of the night more comfortably.

Friday morning.—Complains of excessive weakness, but the general character of the symptoms remains nearly the same. Pulse so intermittent that it could not easily be counted. Accompanied in this visit by Mr. M'Aulay, surgeon, of Lincoln.

Friday evening.—Recurrence of urgent general distress, with great heat of skin, and a full but still intermittent pulse. I bled her, myself, to ten ounces, when she became convulsed, and I of course stopped. She passed a quieter night than usual. Refuses to take medicine.

Saturday morning, (October 18th.)—Complains of throbbing of the temporal arteries, and wishes for leeches. Skin hot; pulse exceedingly intermittent, but very bounding. Applicr Hirudines x. temporibus. Leeches bled well, and gave some relief.

Saturday night.—All the symptoms aggravated. Prays for some relief, and does not object to be bled again. I immediately bled her from the arm, and obtained twelve ounces of blood, but it was a long time in flowing. Remained very faint for an hour afterwards. Great restlessness then supervened, for which I directed thirty-five drops of laudanum, and left her, with little or no hopes of her living through the night. The blood was very watery. Some that spurted off scarcely stained the sheets. Wine and water to be taken at intervals.

Sunday.—Passed a tolerably quiet night. This morning, countenance very anxious; pulse not less bounding, but more regular. Makes no complaint of pain, but says she is very weak. She turns in bed, however, and raises herself when she wants to drink. Thirst very great. Skin cooler than formerly. No stool.

Five grains of Calomel, and a black dose.—Some wine-and-water, beef-tea, and jelly.

Monday, (October 20th.)—Passed a tolerable night. Pulse 108, full, but not intermittent. Anxiety still strongly depicted in the countenance. Says she feels very ill. Bowels freely opened.

Nitre powders every four hours.

Friday, (October 31st.)—Since the last report, the patient has gradually mended, but the alteration in her state from day to day was with difficulty perceptible. The only new symptom has been the expectoration, within the last five days, of a considerable quantity of mucus. The pulse is still quick, but uniform in its beat. Appetite returning; she sleeps well; respiration quite easy; still very weak. Walked across the room yesterday for the first time.

During the two first weeks of November, she suffered most severely from headache, which was relieved by leeches, and ultimately yielded to active purging. Third week in November, found my patient still ailing: very weak; her face was pale and swelled, and she looked like one dropsical; pulse too quick; tongue very foul; appetite improving slowly. She is beginning to do a little work at her needle.

December 2d.—Still poorly, though somewhat better. Goes off tomorrow for Calais, her native place, to try the effect of rest and change of air.

March 1, 1827.—Mademoiselle Anselle is now in Paris, in the enjoyment of perfect health.

*Upper John-street, Golden-square.*

